# ‘See me for me’: An intersectional approach exploring sexual and gender minority medical students' experiences of role models

**DOI:** 10.1111/medu.70185

**Published:** 2026-02-10

**Authors:** Antony P. Zacharias, Robert Douglas, Debbie Aitken

**Affiliations:** ^1^ Department of Education University of Oxford Oxford UK; ^2^ UCL Medical School University College London London UK; ^3^ Lister Hospital East and North Hertfordshire Teaching NHS Trust Stevenage UK

## Abstract

**Phenomenon:**

Sexual and/or gender minority‐identifying (SGM) medical students report lower levels of belonging and heightened discrimination in medical schools, especially among those who hold intersecting identities that are underrepresented in medicine (URM). Role modelling has been identified as a tool to combat this phenomenon. We used an intersectional approach to explore how interacting URM identities in relation to SGM identity mediate the experience of role models to influence feelings of belonging.

**Approach:**

We employed interpretative phenomenological analysis to explore nuanced and heterogeneous role modelling experiences. We conducted semi‐structured interviews with 10 medical students from six medical schools in the United Kingdom.

**Findings:**

Participants described how cisheteronormativity often led to loss of identity control, fragmentation and accompanying self‐inauthenticity to protect their sense of belonging. Intersecting URM identities heightened feelings of otherness, and mediating multiple URM identities was cognitively taxing to many participants, even within traditionally inclusive spaces. Role models empowered participants to reclaim control over their narratives, engage in activism and enact disruptive visibility that challenged hierarchical norms. Participants valued role models who shared their intersecting identities, although many also emphasised that anyone who visibly respected SGM and URM identities could equally effectively foster belonging. Hierarchy and power imbalances prioritised by medicine limited the positive effects of role modelling and perpetuated identity fragmentation and inauthenticity. Accordingly, role models were consistently most visible and positive when in positions of influence. Overall, when positive and available, role modelling relationships provided practical pathways for students to integrate fractured identity threads into a more coherent, authentic self.

**What this paper adds:**

This study is the first to apply an intersectional lens to role modelling experiences of SGM medical students with multiple URM identities in the UK. We offer some practical steps for medical schools to cultivate inclusive role modelling.

## Abstract 2

New paper by @antonyzacharias and colleagues exploring how role models are experienced by medical students who identify as sexual and/or gender minority with other intersecting minority identities. #MedEd

## 1 INTRODUCTION AND BACKGROUND

Health‐care disparities between individuals who identify with a sexual and/or gender minority identity (SGM) and those who do not remain widespread across a plethora of health outcomes,^1^, ^2^, ^3^, ^4^ representing a major public health concern. With recent legislative changes in the United Kingdom (UK) impacting health‐care provision for gender minority individuals, research directed at improving care for SGM communities more broadly has never been more imperative.^5^ Representation of SGM identities in medical schools is one method to improve quality of care, through enhancing patient‐provider understandings mediated by shared lived experiences.^6^, ^7^, ^8^, ^9^, ^10^, ^11^, ^12^ However, belonging remains a challenge for SGM medical students, particularly for those with gender minority and intersecting ethnic minority identities, despite interventions within the curriculum.^13^, ^14^, ^15^ The reasons are multifactorial, including prior experiences of discrimination within and outside of medical training and associated concerns about future career prospects.^12^, ^13^, ^16^, ^17^, ^18^, ^19^ Hence, numerical representation through tick‐box style recruitment is insufficient to truly foster feelings of belonging among students holding underrepresented identities in medicine (URM).

Recognising this, nurturing role modelling relationships has been suggested as a powerful and relatively unexplored tool to foster SGM representation that *already exists* within medical schools.^20^, ^21^, ^22^, ^23^, ^24^, ^25^ Interventions to reduce discrimination among SGM groups in medicine have historically centred on the formal curriculum with limited success.^14^ A focus on role modelling within the hidden curriculum—where learning occurs outside of formal curricula and often through embedded cultures, norms and implicit practices—is therefore prudent.^26^

### 1.1 Theoretical grounding

Role modelling is generally understood as how individuals (henceforth ‘aspirants’) recognise and use qualities others possess and incorporate these into their own practices and sense of self.^27^ The phenomenon encompasses two facets.^28^ Firstly, the ‘role’ is where one recognises a set of behaviours and actions they wish to embody within their social milieu, often based upon their current perception and future aspirations of self. Secondly, the ‘model’ includes the process of identifying a benchmark, especially by those who lead by example, through which one then imagines, visualises, hears and enacts their aspirational role.^29^, ^30^ Hence, role modelling and identity are indistinguishable social concepts; holding a specific identity allows one to relate to models with shared identities, and recognise, imagine, and enact a ‘possible self’ based on past experiences, present circumstances and future desires in their sense of self.^27^, ^31^, ^32^, ^33^ Previous work also highlights the multifaceted nature of role modelling. Notably, negative as well as positive traits can be assimilated (termed negative role modelling), and role models can transcend the individual‐to‐individual level to encompass institutions and collective social spheres which can bear down on an individual‧s sense of self and their aspirations (termed collective role modelling).^25^, ^30^, ^34^

Role modelling has been shown to be particularly powerful for SGM students as this group is more likely to require help navigating the discrimination and adversity they often face in educational settings, especially in the absence of other social support.^35^, ^36^ Our previous work on how SGM medical students specifically experience role models in medical schools highlighted how role models are an essential tool to improve their sense of belonging.^25^ However, we could not fully capture how *multiple minority identities intersect* to influence the role modelling dynamic. Intersectionality theory, first coined by lawyer and social activist Kimberlé Crenshaw in 1989,^37^ continues to be used broadly to explore how those holding varied identities which are discriminated against interact to uphold power imbalances and inequities in societies within which they are embedded.^38^, ^39^ Intersectional approaches are crucial in this area, as SGM individuals are more likely than non‐SGM counterparts to hold other minority identities.^40^ For instance, East Asian SGM medical students are less likely to be ‘out’ than white counterparts,^16^ and others reiterate how multiple intersecting URM identities create a ‘double penalty’ of heightened discrimination, identity sacrifices, and difficulty belonging within medical school.^9^, ^17^, ^21^, ^41^, ^42^ Accordingly, the lack of URM representation within medical schools means SGM—and particularly more visible gender minority—medical students from non‐white ethnic backgrounds recognise a greater need for role models across intersectional identities to foster belonging in medicine.^42^ Hence, exploring how role models are experienced by a range of URM identities is more pertinent than ever, especially as increasing proportions of students from a range of URM backgrounds are entering medical schools.^43^

Our previous work also demonstrated that positive role modelling relationships were significantly impeded by cisheteronormativity.^25^ Centring the ways that cisheteronormativity—the prioritisation and normalisation of straight, gender‐conforming identities within society—influences role modelling relationships is vital to accurately understand and reflect participants' experiences. Consequently, we engage with Queer Theory throughout this work, whose foundational paradigm is that cisheteronormativity is perpetuated and sustained through systems of power inequities and oppression. Through examining participants' experiences through this lens, we can begin to foreground and challenge the underpinning structural processes that uphold cisheteronormativity, with real‐world application.^44^, ^45^ Intersectional approaches fit neatly with this paradigm, as both seek to understand how and why minority experiences are maintained as non‐normative through systems of oppression and power.^39^, ^46^, ^47^ Consequently, the three main frameworks we use in this work (Queer Theory, intersectionality and role modelling) are complementary to explore how often implicit but powerful agents may influence cisheteronormativity and therefore mediate role modelling relationships in medical schools.

### 1.2 Research direction

Taken together, we sought to explore how medical students who hold both SGM and other intersecting URM identities experienced role models, as a tool to improve their sense of belonging in medical school. Examining their experiences at the intersection between URM and SGM identities is an approach recognised to be lacking in medical education,^19^ and allows for a more nuanced and real‐world exploration of role modelling, which without specific attention is often implicit and hard to otherwise foreground.^42^, ^48^ Our research question was ‘how do experiences of role models influence the sense of belonging for SGM‐identifying UK medical students, and how do their other intersecting URM identities influence these experiences and sense of belonging?’

## 2 METHODOLOGY AND METHODS

### 2.1 Methodology and analytical approach

**FIGURE 1:**
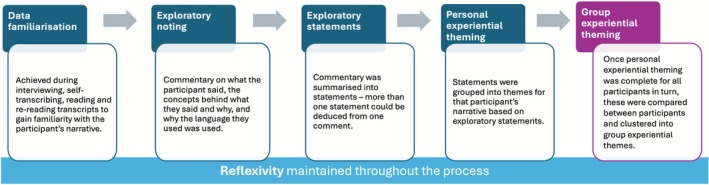
The process of interpretative phenomenological analysis (IPA) used, with reflexivity positioned throughout the process to maintain analytical validity. The purple colour used in the last step is the only point where themes between participants are compared, meaning IPA allows the preservation of idiosyncratic, heterogenous experiences in ways other methodologies may not. [Color figure can be viewed at wileyonlinelibrary.com]

Given the heterogeneity between and within URM experiences, we required a qualitative analytical method that would respect diversity within each narrative.^49^ In addition, some intersectional approaches have previously homogenised experiences further as identities are often compartmentalised into socially ascribed categories.^50^, ^51^ Interpretative phenomenological analysis (IPA) is well suited to our aims as it values difference by seeking to interpret meaning from, and make sense of, an individual's *unique* experiences,^52^, ^53^ rather than finding commonalities in narratives that other methodologies aim to achieve.^54^ Further details on the IPA process can be found in Figure 1. Its application to Queer Theory perspectives is also suited, given that the meaning‐making element of the approach allows us to more closely interrogate how implicit power structures exert influence over SGM identities in medical school.^55^

Of note, it remains conceptually difficult to talk about SGM experiences without referring to the non‐SGM ‘other’, especially as this is often how participants described their experiences. Consequently, we refer to SGM and non‐SGM individuals/groups for illustrative purposes and underscore identity fluidity specifically when mentioned by participants to mitigate binary discourse as far as possible.

### 2.2 Reflexivity

Reflexivity is essential to consider with both IPA and intersectionality research as we, as researchers, are actively interpreting participants' experiences in the context of our own biases, worldviews and intersectional identities—termed ‘double hermeneutics’.^47^, ^53^ I (APZ) identify as a clinician who is a homosexual cisgender White man, holding an intersecting refugee and religious minority identity. These have influenced my own experiences navigating my identity personally and professionally, greatly limiting my identity expression. I became interested in how role models can alter the experiences of SGM‐identifying individuals after reflecting upon their utility to me through both medical school and my career so far, having only relatively recently become more open in my identity. I (RD) identify as a gay cisgender White male clinician with a hidden mental illness. As someone who had few role models in medical school, I felt drawn to this project to hopefully help understand and develop ways to enable students to feel a sense of belonging through role modelling to which I did not have access. I am relatively new to qualitative research and approached the project with some trepidation both in its process as well as appreciating my relative privilege as a White male. As such, I was nervous when interpreting transcripts to not misconstrue phenomena particularly related to minority gender and ethnicities to which I have little relational experience. However, I felt reassured by the member‐checking elements of the project to ensure alignment of my interpretations with participants' lived experiences. I (DA) have dedicated much of my medical education career, including research, to social justice issues, particularly related to supporting SGM students and staff. As a queer White woman with unseen disabilities, my lived experience, including navigating homophobic and ableist microaggressions, deeply informs my approach to interviewing and data interpretation. Additionally, my previous experiences as an SGM‐identifying senior member of staff in medical schools further shape my perspective. I remain mindful of how my identities and senior role may influence interactions and analysis, actively reflecting on these dynamics throughout the research process.

We kept reflexive journals during our respective participant interviews, and during the analysis and synthesis of data. We remained mindful throughout the process that each person's multiple intersecting identities weave a unique canvas of experiences that cannot truly be appreciated by anyone else, even as researchers. However, we feel that through contemporaneous and retrospective member‐checking, and drawing upon our own experiences as URM individuals navigating medicine and medical education, we were able to give as close to a valid voice for our participants as possible.

### 2.3 Participant recruitment

We recruited participants between February and April 2025 using purposive sampling because of the identity‐specific nature of our research question, following ethical approval by the University of Oxford Departmental Research Ethics Committee. Information sheets and advertisements were distributed to all UK medical schools' education leads for further cascade via the Medical Schools Council following approval by their committee, in addition to student society bodies. Interested participants then completed a consent form, followed by a demographic survey hosted on Microsoft Forms© to determine eligibility. Socioeconomic status was assessed using a combination of postcode at time of medical school application, annual household income, level of parental education and type of schooling attended. URM identities are relational and contextual; although certain identities may not be numerically underrepresented at medical school, feelings of otherness and subordination are highly individual.^51^ As such, we encouraged free‐text description of URM identities to capture these individual experiences of minoritisation. Similarly, Queer Theory underscores the contextual and temporal fluidity of SGM identities; we ensured participants could self‐describe their SGM identity to avoid fixed categorisations as far as possible.

Participants were eligible for interview if they fulfilled each of the following:

- being a current medical student at a UK medical school,
- holding at least two URM identities, one of which being an SGM identity, and
- consent to participate in the interview.

### 2.4 Data collection and analysis

We aimed to interview 10 participants, as smaller participant numbers with IPA approaches allow for a more nuanced exploration of experiences.^56^ We pseudoanonymised participants with a code ‘MS’ followed by a number denoting their order of consenting. We (APZ, RD and DA) conducted one‐on‐one online semi‐structured interviews using Microsoft Teams, allowing us to deeply and dynamically explore each participant's experiences of role modelling in turn. The interview schedule was developed following an extensive literature review of the current understandings of both role modelling and intersectionality (summarised in Section 1). APZ piloted the interview schedule on an SGM‐identifying former medical student with a chronic mental health condition. From this, only one question required adjustment (asking about role modelling implicitly to prevent biased role model identification, before explicating it in a later question) (Appendix S1).

Interviews were transcribed by the interviewer to ensure immersion in the data and to align our contemporaneous interpretations with the analysis. We transcribed verbatim interruptions, utterances and pauses to capture experiences buried within linguistic and discursive nuances. When noting, a ‘role model’ was considered when experiences included individual or collective bodies who influenced participants' internal and external identity, possessed qualities that participants strove to emulate or vice versa, and/or where participants themselves explicated role modelling. We used Microsoft Word© for all analyses, following the stages outlined in Figure 1. Each interview was analysed in sequence directly following data collection to ensure initial interpretations were accurately captured and that data immersion was as high as possible. We sent each participant a summary of their analysis to ensure our interpretations spoke to their experiences.

## 3 RESULTS

Fifty students consented to participate, with 38 completing the demographic survey. We recruited 10 participants across six medical schools, selecting participants with varied SGM and intersectional identities to ensure a broad range of experiences were captured. A summary of participants is given in Table 1 for context.

**TABLE 1:** Participant demographics stated for contextual grounding of data in this section.

Participant ID	Region of university	Year of study	Sexual identity	Gender identity	Intersectional minority identities
MS1	Scotland	6	Bisexual	Agender	Neurodivergence
MS2	Northwest England	5	Lesbian	Cisgender woman	Neurodivergence; low‐income background
MS3	London	5	Queer, demisexual/asexual spectrum	Queer/genderqueer	Neurodivergence; religious minority
MS5	Southeast England	5	Queer/bisexual	Genderqueer	Ethnic minority (second generation immigrant); hidden disability; chronic mental health issue
MS8	Southeast England	4	Homosexual/queer	Non‐binary transmasc: androgenous presentation, assigned female at birth	Ethnic minority; chronic mental health issue; low‐income background
MS9	Southeast England	6	Homosexual	Cisgender man	Ethnic minority (mixed heritage); religious minority; chronic mental health issue; neurodivergence; parental estrangement
MS13	Southeast England	6	Lesbian	Non‐binary	Neurodivergence; chronic mental health issue; hidden disability; lower socioeconomic background
MS15	West Midlands	3	Bisexual	Cisgender woman	Ethnic minority; neurodivergence; low‐income background
MS25	Southwest England	2	Queer with a preference for women	Cisgender woman	Ethnic minority; chronic mental health issue; parental estrangement
MS33	Scotland	1	Queer	Genderfluid	Ethnic minority; lower socioeconomic background

*Note*: We have stated identities as written by participants to preserve nuances, although condensed in places for tabulation. We have not included specific ethnic and/or racial minority details as many participants had mixed heritage backgrounds, where stating these alongside other presented demographics risks participant identification.

We deduced four key themes that spoke to the ways that the intersection of SGM with other URM identities influenced the experience of role models and associated feelings of belonging. A summary is given in Table 2, which serves as a map for our results. We have edited quotes for clearer presentation only where this does not disrupt the linguistic elements of the analysis. We visually set out the commonalities and differences between participant experiences within each theme and subtheme in Appendix S2.

**TABLE 2:** Overview of the four group experiential themes, sub‐themes and illustrative data. This serves as a map for the detailed analyses set out in this section.

Group experiential themes	Sub‐themes	Illustrative data
Identity fragmentation, conflict, negotiation and inauthenticity *Experiences of authenticity to their intersecting URM identities, the relationships between them, and how they navigate maintaining an authentic ‘whole’ self*.	A nexus of identities and self‐authenticity Inauthenticity and internal identity conflict Towards an authentic self	‘I think it's always nice to have/see people in the world who [have] similar traits to you […] I think the way it's worked out is that my communities have ended up becoming separate in terms of parts of my identity and I'm kind of like the only intersection that I know of.’ (MS25; lines 498–501)
Negotiating a sense of belonging at the margin *Experiences of marginalisation because of their intersecting URM identities, and how this is navigated, mitigated, and the process of moving closer to the centre of the medical school community*.	Compounding experiences of ‘otherness’ Shared minority experiences in role modelling relationships Defining new margins: communities and belonging	‘[Medics theatre has] always been a really queer space […] We have a lot of bi people and lesbians so maybe not like the same identity but they've always been very very supportive […] I think the queer community is so expansive that I think you're probably not going to find somebody that necessarily matches but it's about having that kind of space.’ (MS3; lines 312–320)
Making it ‘their’ problem: carrying the minority burden *Narratives of fear and mental taxation that holding multiple URM identities creates in addition to SGM identities*.	Effort and uncertainty within normative contexts Towards alleviating the minority burden Visibility and possibility in unburdening identities	‘It takes one person to understand your pronouns and respect them and then for that one person to essentially model that across a professional team for it to take […] It's made me much less scared to come out to people.’ (MS8; lines 518–522)
Identity ownership and the path to self‐actualisation *Experiences of loss of identity control with compounding URM identities, mediated by normativity, and how role models are utilised to take back power over identity ownership*.	Normativity and identity control Contextual agency in reclaiming identity ownership Empowerment, activism and disruptive visibility	‘I just felt like that the way that session was designed … it felt like it had not been designed by someone who has actually experienced that before. And yeah I wrote a long sort of essay of feedback […] I've definitely noticed things that probably some of my other colleagues wouldn't have noticed or wouldn't have been affected by.’ (MS9; lines 208–221)

### 3.1 Theme 1: identity fragmentation, conflict, negotiation, and inauthenticity

#### 3.1.1 A nexus of identities and self‐authenticity

Many participants described their SGM identity as ‘encompass[ing] quite a lot of different identities’ (MS25; lines 4–5), nested within and between wider intersecting selves, and often central to the way other identities are experienced. MS3 described their SGM identity as fluid between ‘bits of my life where I hold it … stronger, or even where I want to hold it more strongly’ (MS3; lines 28–29), but that cisnormativity means displaying SGM identity is not something that they ‘know if I like that or not’ (MS3; line 35). MS5 also described this phenomenon, where the revelation of their SGM identity might have professional repercussions, so forces the foregrounding of their disability despite recognising that both identities are equally intrinsic to an authentic whole self in their ‘real world’ outside of medicine (MS5; line 533). Their experiences indicate a commonality between many participants: ‘trying my best to be authentic to myself’ (MS8; line 535), but feeling conflicted, restricted and ultimately defeated because of the overbearing power of cisheteronormativity and associated discrimination across a range of their URM identities.

#### 3.1.2 Inauthenticity and internal identity conflict

In multiple experiences, normative contexts within medical school define identity and force unnatural identity fragmentation. Participants described having to split into multiple ‘versions of [myself]’ (M13, line 497) to conform to normativity and fit in. MS5's use of the phrase ‘struggle Olympics’ (line 123) when describing their identity nexus also evokes a sense of competition between URM identities, where there is only space for one in medical school. The fragmentation of identities can increase feelings of self‐inauthenticity and associated conflict between ‘want[ing] to try and be myself without fear of judgement’ (MS25; lines 265–266) and it being ‘a good thing for me’ to avoid discrimination through identity concealment (line 131). Consequently, the ability to ‘hide [SGM identity] when I need to’ (MS25, line 119) as a protective mechanism in normative environments is viewed as a ‘massive privilege’ (MS3; line 42) for many, even at the expense of authenticity and associated identity conflict.

#### 3.1.3 Towards an authentic self

Role models were a tool in many participants' narratives to begin to view oneself as unified across intersectional identities and authentic to each. Corroborating this, a lack of role models was attributed by some as maintaining identity conflict, leading to a desire for role models to showcase the potential of authentically displaying all their minority identities in medical school.

*‘I think one of the biggest reasons why I have such a hard time properly coming to terms with my identity is just that*, *that I don't know any other brown people who are like*, *queer […] I wish there was a sort of cross section basically because I/I know I'm/I know I'm not the only one*.’ (MS25; lines 375–381)

MS13 reiterated this, describing how role models sharing their socioeconomic identity are central to their feelings of belonging and heighten their desire for role models across their SGM and wider URM identities.

### 3.2 Theme 2: negotiating a sense of belonging at the margin

#### 3.2.1 Compounding experiences of ‘otherness’

Most participants felt that their multiple minority identities, coupled with a lack of visible others sharing those identities, mean ‘you're maybe represented in one sense but maybe not in another sense … in the sense that [multiple minority identities] compound’ (MS5; lines 178–179). This can intensify experiences of discrimination and feelings of being ‘isolated … a bit out of place’ (MS1; line 180) from peers. For instance, the compounding of MS9's lived experience of stigma in his religious and sexual identity heightens feelings of non‐acceptance by both communities; therefore, exacerbating his experience of otherness within both spaces.

*‘I'm aware that a good chunk of [my religious group] are not homophobic or hold sort of more nuanced views around LGBTQ+ people [but] it's almost safer to assume that they're homophobic until proven otherwise*.’ (MS9; lines 118–120)

#### 3.2.2 Shared minority experiences in role modelling relationships

All participants described looking to role model figures as examples of belonging in a context that is otherwise othering.

*‘Being able to kind of look at them and go*, *OK*, *I'm not going to be the only person in the world who's in that position […] you feel like no matter where you go*, *you're going to be like the only one in this environment and kind of knowing that there are other people*. *I think that's kind of what would make a role model for me*.’ (MS13; lines 597–605)

To maximise feelings of belonging through role modelling, many participants highlighted the importance of matching across intersectional identities, despite all participants recognising a lack of matched role models. The degree to which complete matching was desired was variable. On the one hand, MS33 underscored that they aren't ‘fitting in as much as other queer people [because] sometimes it's a race problem’ (lines 354–355), where identity‐matched role models with shared lived experiences would allow them to be ‘on the same wavelength’ (line 355). As such, some participants (MS1, MS8, MS9, MS33) described either turning to online role models who shared their multiple identities, or amalgamating identities from various sources to apply to their own intersectional experience through ‘seek[ing] role models from many different backgrounds’ (MS8; line 488).

On the other hand, many (MS2, MS3, MS5, MS8, MS9, MS13, MS15) felt perfect matching across intersectional identities is either unrealistic or not necessary. Some participants (MS2, MS3, MS5, MS15) stated that simply being ‘a bit … down to earth and just [taking] you as you’ (MS2, lines 325–326) can be enough to role model a sense of belonging. Similarly, MS2 and MS9 expressed that seeing those from other minority groups still allows them to ‘gain hope and reassurance’ (line 381) without being matched in identities, underscoring that perceived commonality in experiences across URM identities can be enough to facilitate role modelling and associated belonging.

#### 3.2.3 Defining new margins: communities and belonging

Where individual role models could not readily be identified, many described agency in selectively associating with communities to position themselves from the margins to the centre. These ‘network[s] or communit[ies]’ were often described as bolstering ‘solidarity’ (MS5, lines 137–142) with other SGM‐identifying peers. Role modelling qualities were highly prevalent within respective communities, even if not explicitly stated as such:

*‘The queer medics club [is an inspiration] just because of the way they showcase themselves and their pride uh at being who they are. And I guess being authentic in themselves’* (MS33, lines 467–468)

Some participants identified individual role models through Queer‐specific events and communities (MS1, MS13, MS25, MS33), whereas all participants alluded to role modelling also being a more nebulous and collective process, where being surrounded with ‘a good community of people where there was no “oh that's so weird, you haven't got a boyfriend or something”’ (MS25, lines 67–68) heightens feelings of belonging and self‐identity projection within that chosen space.

Similarly, all participants described how collectivisation of individuals facilitates a greater degree of power and influence over cisheteronormative contexts than might be possible alone. As such, role modelling processes were evident not just within interpersonal interactions but also through wider environmental or contextual factors, disrupting normativity and role modelling the possibility to be visible: ‘people start coming out as gay, and then it's cool to be gay, and it's a sort of huge social 360’ (MS5; lines 263–264). MS3 also stated that they hope that their influence as an individual role model themselves might ‘trickle down into the culture a little bit’ (lines 453), underscoring the collective, albeit unpredictable, power role models may have over contexts as well as individuals.

However, MS33 reiterated that even within Queer spaces, diversity can be limited and stifle feelings of true belonging: ‘A lot of Queers are also white, and it's just, there's not as much representation, even in the queer medic's club’ (MS33; lines 378–379).

### 3.3 Theme 3: making it ‘their’ problem: carrying the minority burden

#### 3.3.1 Effort and uncertainty within normative contexts

All participants described mediating multiple identities in terms of safety and uncertainty, influenced by normativity. Safety was considered not only in terms of overt discrimination but also fear of social isolation and biased professional progression. The relationship between imbalances of power and hierarchy valued by medicine (for example, between students and consultants) and the safety of SGM disclosure was a theme identified for all participants.

‘*I don't know if I feel safe if senior doctors are aware of [my minority identities] […] we're in the middle of an operating theatre and suddenly [people who have noticed my badge] suddenly go “I've just noticed that like*, *you're queer” and it's like “yes*, *and the consultant over there is my firm lead, please shut up*.”’ (MS3; lines 36–39)

Some participants reiterated that their multiple intersecting URM identities existing in a normative environment leads to ‘second guess[ing]’ (MS2; line 272) and ‘doubting your own judgement’ (MS1; lines 402–403) about which URM identities, if any, contribute to their negative experiences in medical school. For instance, MS2 described an experience where she was actively excluded from an educational opportunity by a faculty member and reflected that ‘if instances do happen […] it isn't ever explicitly mentioned but it's always kind of in my head whether [being visibly gay and/or autistic] did play a part.’

The uncertainty around safety and discriminatory experiences meant all participants felt they were guessing how to ‘moderate that disclosure […] it's also something that you then have to think about’ (MS3; line 43). Many described lacking ‘the resources available mentally’ (MS25; line 175) to do so, leading to mental exhaustion and feeling ‘defeated’ by the burden of identity mediation (MS8, line 216). Compounding this, a lack of role models to show participants the possibility of acceptance within medicine meant many participants felt alone in carrying the burden ‘to make sure that my identity is respected’ (MS8; lines 206).

#### 3.3.2 Towards alleviating the minority burden

Although some described the power differentials between faculty and students as heightening feelings of unsafety in SGM identity disclosure, many participants also recognised not only the role but also the responsibility that those in positions of power—namely faculty and their associated collective *influence* as ‘the medical school’—have in lifting the minority burden in normative contexts. For instance, some (MS2, MS15, MS33) recognised that within the power imbalance between faculty and students lies associated legislative responsibilities, which can create a safety‐net to lift part of the minority burden: ‘it's a professional relationship where they kind of … can't be discriminative of me, legally’ (MS2; lines 43–44). Hence, the imbalance of power between faculty and students can paradoxically facilitate easier disclosure of SGM identity.

Many participants (MS8, MS9, MS13, MS15, MS33) reiterated that those in positions of power have the greatest influence, and hence duty, to alleviate the isolating burden they feel, through role modelling the welcomed presence of SGM identities in medical school. This narrative was especially prominent for MS8, where they recognised that ‘it takes one [senior person] to understand your pronouns and respect them and then for that one person to essentially model that across a professional team’ (lines 519). By those in power taking the onus, it means the burden is ‘on everyone else’ (line 234) and not resting solely on their shoulders.

#### 3.3.3 Visibility and possibility in unburdening identities

Role models remained an important tool in participants' narratives to foster a sense of possibility in showcasing *all* intersecting identities, demonstrating that ‘people like her exist […] maybe I can feel like that one day’ (MS2; line 341). By doing so, role models can show that SGM medics holding wider URM identities ‘are here and can be visible’ (MS8; line 281) in the face of normativity. For this effect to be most powerful, all participants identified that clear visibility is needed to not only identify role models but also for those potential role models to actively demonstrate comfort of their identities belonging, rather than just existing, in medicine.

*‘It's nice to see that there are other professionals who I sort of look up to as like medical role models who are able to talk [their identities] very openly and feel comfortable sharing that on a stage in front of like 180 medical students*.’ (MS9; lines 361–363)

Participants recognised that moments in which their own SGM identity was most visible was when they considered themselves a potential role model also. This often involved giving other students advice on navigating medical school while holding SGM identities. Hence, visible role models can relieve students of some of the burden of identity suppression, fostering authentic identity visibility, which in turn perpetuates a cycle of further possible role model visibility and utility.

### 3.4 Theme 4: identity ownership and the path to self‐actualisation

#### 3.4.1 Normativity and identity control

All participants' experiences contained narratives around loss of control of their SGM identity, including when and how their SGM identity was prioritised, expressed, and interpreted. For instance, MS15 described how normative societies place heightened significance on her sexual identity when ‘a woman liking women should just be normal’ (line 548), without others needing to define its importance in her own life. Power imbalances amplify this, largely because of the inability to retaliate against the process. MS8 exemplified how it is hard to regain control over the use of their intentionally wrongly‐used preferred pronouns as it is perceived to be ‘detrimental or awkward to correct […] the consultant or a senior’ (lines 127–128) because of the power differentials at play.

#### 3.4.2 Contextual agency in reclaiming identity ownership

Accordingly, those in positions of power, especially faculty, were perceived to have greater control over contexts, and, as role models, allow all participants to feel that they could reclaim ownership over aspects of their identities. As such, they are more able to ‘[create] a good environment’ (MS5; line 363) and ‘[set] an example’ (MS13; line 189) to which others listen, beyond what participants feel able to do themselves. Role modelling in this regard works beyond individuals, with institutions also recognised as holding power to role model comfort in SGM identity—another example of ‘collective’ role modelling:

*‘A lot of NHS Trusts have dedicated LGBTQ campaigns or celebrate things like pride month. All that sort of helps me to feel reassured that being gay will not be an obstacle hopefully in my career in the future’* (MS9; lines 350‐352)

#### 3.4.3 Empowerment, activism and disruptive visibility

A strong theme running through all participants' experiences was one of activism in challenging existing powers that maintain normativity, as well as empowering others through role modelling. Some (MS1, MS5, MS8, MS9, MS25, MS33) utilise relatively understated forms of activism, for example, symbols like lanyards and badges, to outwardly but subtly challenge the absence of SGM people within medicine. For others, activism takes on more direct forms through written complaints (MS9), challenging normative comments (MS1, MS2, MS33), or taking on pastoral roles within URM‐related societies (MS25). MS5 recalled that peers that ‘questioned things that have been said’ are people they ‘look up to’ (lines 381–382), reiterating that peers who challenge power differentials showcase activism as possible, as well as allowing greater visibility of potential role models.

Activism was viewed by most participants as an intrinsic quality held by people with multiple URM identities. MS3 summarised their identity as the ‘practising of sexuality and gender that are not normative, that might be challenging ways sexuality and gender have historically been “done”’ (lines 17–18). ‘Practising’ emphasises the active and purposefully disruptive nature of holding SGM identities for them. Additionally, multiple URM identities sensitise many participants to wider moments of discrimination across other minority identities and accordingly heightens the responsibility many participants feel to advocate for others in moments of injustice (MS1, MS2, MS9, MS13, MS33).

However, some still feel that ‘as a junior person especially, it can certainly be difficult’ (MS1; lines 404–405) to retaliate against pervasive power imbalances in medicine, where hierarchy is ingrained and heavily valued. This is especially difficult for participants (MS13, MS33) who were more ‘mild mannered’ (MS13, line 122) as they feel less able to challenge normative powers, despite seeing peer role models who are ‘very confident, very vocal, very good at combating things’ (MS13; lines 573).

Additionally, with SGM identity being ‘something we “do” rather than something that just exists’ (MS3; lines 18–19), MS8 described feeling ‘a lot of pressure in being the first “X” that someone has ever met […] like I'm representing my whole community when really I can only represent myself’ (lines 548–550). MS8's experience builds on concepts in Theme 3 by reiterating the mental burden that being a potential role model and associated activism intrinsic to the role can bring.

## 4 DISCUSSION

**FIGURE 2:**
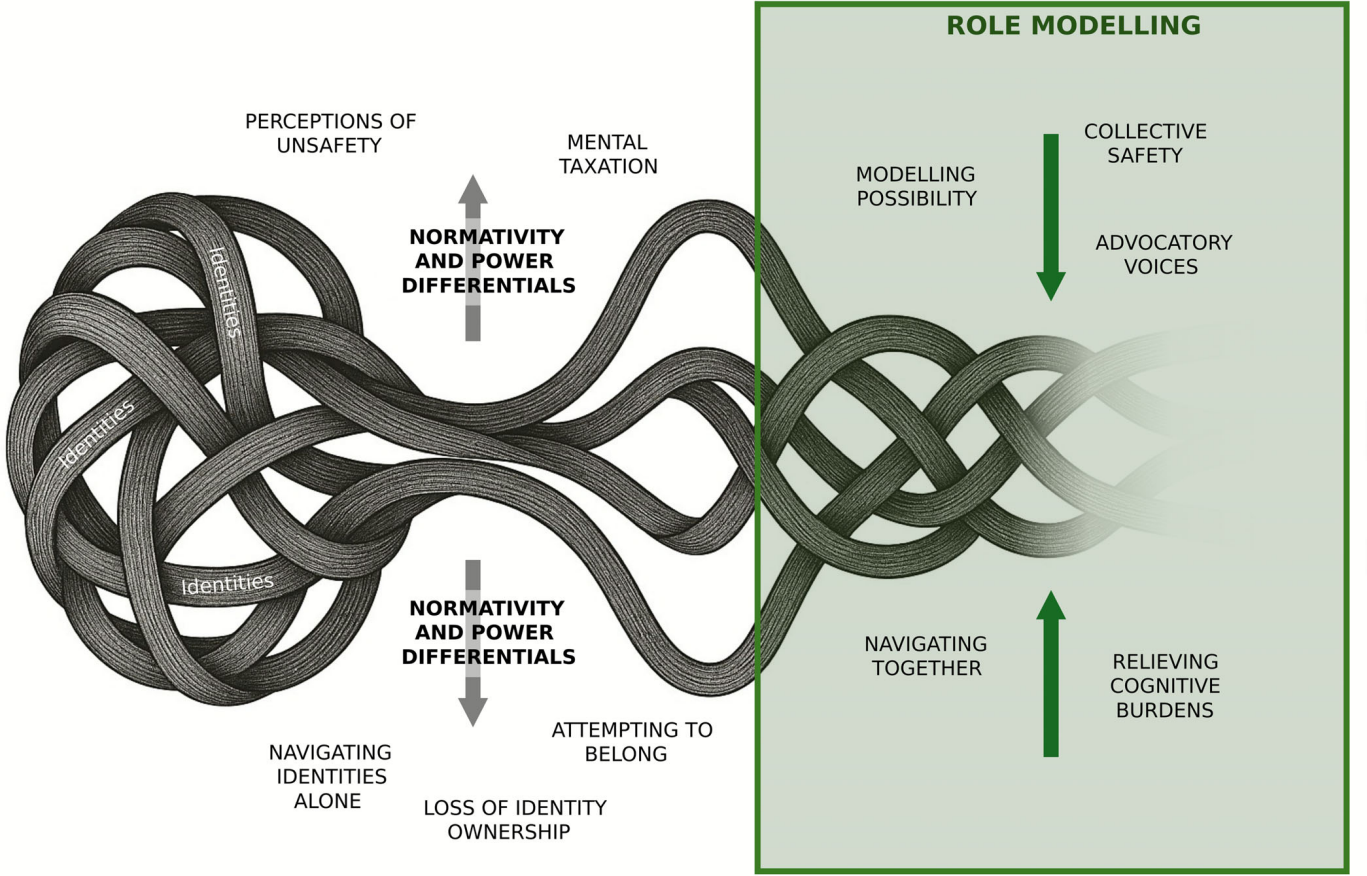
The threads of one's identity and multiple intersections are mediated by the contexts within which one exists. Normativity, exerted across minority identities, tends to separate out the threads of identity. The identified drivers and effects of this are given. This process is heavily mediated by power imbalances which uphold normativity. Role models can begin to show how an individual can re‐thread their multiple identities back into an authentic whole self. The factors that assist this are given within the green box symbolising both collective and individual role modelling relationships. [Color figure can be viewed at wileyonlinelibrary.com]

By employing the analytical lens of intersectionality alongside Queer Theory paradigms, we were able to explore the complex experiences that some SGM‐identifying medical students have with role models, and how these role modelling relationships are altered because of intersecting URM identities. We deduced a common narrative from most experiences. Participants described existing with multiple minoritised identities in medical school—a context perceived to be highly normative. Minoritisation and subordination of wider visible URM identities, compounding with cisheteronormativity, intensified the need to artificially fragment identities. Power differentials were a large perpetuator of identity fragmentation and lack of identity control. As such, participants often rendered parts of their selves invisible in order to help preserve feelings of belonging; a phenomenon also described in other works.^9^, ^42^, ^57^ Many described these identity conflicts and associated inauthenticity as isolating and tiring to mediate. However, most also underscored a political and advocatory drive intrinsic to SGM identity, meaning most participants felt an obligation, if not a strong desire, to push against the powerful normative forces that historically subordinate their multiple URM identities. Role modelling qualities were identified throughout this narrative. Namely, role models helped: display the possibility of reintegrating and projecting authentic whole identities into normative spaces; ameliorate feelings of social and psychological isolation through lessening the ‘minority burden’; and allow participants to rebalance the power differentials that suppress their whole integrated identities through becoming activists to challenge normativity. Figure 2 diagrammatically represents these processes that underpin the fragmentation and reintegration of SGM with wider URM identities, underpinned by power differentials, (cishetero)normativity and role modelling.

Participants themselves were also most obviously role models for others when taking on activist roles. Accordingly, our findings underscore that tokenistic representation of singular identities is not enough.^21^ Individuals holding a *range* of URM identities in medical schools may facilitate more powerful role modelling through demonstrating the comfortable existence of whole authentic identities in spite of medicine's normativity.

### 4.1 Role models in challenging power differentials

Power imbalances and hierarchy wove a thread throughout the narrative of all participants. Although our previous study also identified power differentials hindering positive role modelling relationships,^25^ its influence was felt more keenly here. This may be because those holding multiple URM identities experience the effects of subordinating normativity across a greater range of their identities, and more frequently, compared with those only holding an SGM identity.^58^ Medicine's ongoing preoccupation with graded hierarchies reinforces differentials in power, which can disproportionally affect those with multiple URM identities who hold less social and mental capital to challenge normativity alone compared with peers.^59^, ^60^

Accordingly, like in previous works,^15^, ^61^, ^62^, ^63^ participants often explicitly looked to those who were perceived to have greater power over medical school contexts—faculty—as role models to showcase the possibility of SGM and wider URM identities existing in medicine. However, more implicitly, role modelling was also experienced through the collectivisation of SGM identities either physically in Queer spaces such as clubs and societies, or conceptually through institutional cultures. Participants often felt greater safety and empowerment in pushing against the power imbalances as a collective force than doing so independently, lessening the associated ‘battle fatigue’.^60^ The power of collective role modelling is reiterated in other works exploring URM medical students' belonging.^11^, ^25^, ^64^

Although power dynamics controlled and defined many participants' experiences of their multiple identities, they simultaneously heightened most participants' volition, or even obligation, to advocate both explicitly and implicitly as role models for others who shared minority experiences. While this resistance against normativity has been described in related literature on professional identities,^58^, ^60^ our results demonstrate this phenomenon occurring across personal identity intersections also. Fostering this resistor identity in SGM medical students can not only facilitate grass‐roots social justice in the face of pervasive normativity^65^ but also enable the role modelling process through easy role model identification.^25^ However, some participants' experiences highlighted a ‘minority tax’ bestowed upon them to innovate, educate, and advocate for minority groups.^66^ This additional burden of identity activism and associated role modelling may just be another price to pay towards their minority tax.

The duality of advocatory role modelling occurring both for and by SGM students reinforces the ‘double funnel framework of role modelling’ introduced in our previous work.^25^ The framework sets out a bidirectional process of how internal perceptions of identity on one end, and external contextual influences on another, converge onto an individual to influence identity projection, mediated through role modelling. Within this framework, our present study shows how advocacy, heightened by intersecting URM identities, can reverse the traditional trajectory where hierarchical top‐down institutional change alone is expected to foster inclusion. When SGM students are empowered, active and subsequently visible as role models, pervasive normativity can also be challenged from the grassroot student body and catalyse further role modelling opportunities.

### 4.2 Shared experiences in the role modelling relationship

The extent to which participants felt role models were required to share all their URM identities was variable. Although many did feel that mirrored identities would allow them to see the possibility of their entire identity existing in medicine, others felt this was either unrealistic or unnecessary. The literature reflects these conflicting views. On the one hand, many show provision of a higher standard of care and advocation for URM health‐care users when matched identities exist between health‐care provider and user.^6^, ^8^, ^9^, ^10^, ^11^, ^12^ Accordingly, a recent scoping review demonstrated that role models matched across intersecting identities were most powerful in their effects,^67^ although included studies were not limited to medical education contexts. On the other hand, some have shown identity matching is not required for positive role modelling to occur for SGM‐identifying medical students^41^; rather, shared experiences of discrimination across URM identities may be enough.^68^ In accordance with other work,^57^, ^69^ we found that most participants valued role models who were open and accepting of URM identities regardless of the identities those role models held themselves. Our findings suggest that all faculty and medical student colleagues have the obligation to enact positive change through simple actions to be identified as a positive role model. This can be achieved by use of terminology (e.g. using gender‐neutral pronouns), symbols (e.g. ‘pride’ badges and lanyards) and actively reflecting personal identities in professional spaces. Engaging those who are traditionally seen to uphold normativity as allies rather than oppressors, through fostering positive role modelling relationships, may also help to dampen the ‘minority tax’ SGM individuals face.

### 4.3 Applications to practice

Table 3 presents actionable potential interventions based on our findings. As previously discussed, our participants more keenly felt the heightened effect that power differentials played on role modelling processes because of their multiple minority identities (Figure 2). Consequently, interventions must facilitate those from intersectional SGM and URM identities to *reclaim* the power over their identities, and hence agency within medical education spaces. Although we identify discrete interventions from each theme, it is important to recognise that any effort that gives voice to URM medical students can have cross‐cutting implications across themes through simultaneously reducing perpetuating power differentials, facilitating belonging and self‐actualisation, and alleviating the minority burden via role modelling processes or otherwise.

**TABLE 3:** Suggested actions for medical schools to foster belonging of SGM‐identifying medical students holding other minority identities through role modelling, together with possible short‐ and long‐term impacts.

Context	Themes identified	Where it happens most	Example action and safeguards	Immediate outcomes	Long‐term outcomes
Marginalisation of SGM individuals holding other minority identities	Theme 1: Identity fragmentation, conflict, negotiation, and inauthenticity Theme 2: Negotiating a sense of belonging at the margin	Clubs and societies; educational space; social spaces	Visible representation of SGM identities with other URM identities held by people in power Representation through specific educational sessions, and encouraging discussions around identity and personal circumstances in medicine	Role modelling of possibility for those with URM identities Safety to express identities more freely	Stronger, bidirectional role‐modelling relationships; improved equity culture
Hierarchical cultures maintain power imbalances felt most by URM students	Theme 3: Making it ‘their’ problem: carrying the minority burden Theme 4: Identity ownership and the path to self‐actualisation	Clinical placements; supervisory relationships; assessment teams	Co‐creating practices with student bodies as opposed to top‐down policy or curricular implementation Encouraging and safeguarding the student voice, e.g., on committee boards; real‐time feedback channels Representation of SGM identities in leadership who can relate to lived experiences of students, with safeguards for these faculty Peer‐to‐peer assessments that can contribute as meaningfully to progression as faculty‐to‐student assessments	Fairer interactions in day‐to‐day learning and assessments Instilling a meaningful sense of agency among the student body	Reduced systemic power imbalances through flattening of unhelpful hierarchies Facilitation of bi‐directional role modelling
Implicit moments of role modelling occur through language, symbols and discourse across minority identities	Theme 4: Identity ownership and the path to self‐actualisation	Small and large group teaching sessions; clinical environments	Training delivered to both students and faculty/clinicians on their implicit influence through use of language Distributing and encouraging visibility of badges/lanyards, etc. for staff and students to wear, regardless of their own identities Accountable, responsive and confidential reporting mechanisms with meaningful action taken to moments of microaggressions or overt discrimination Distributing initiatives and workload around equality and belonging away from solely SGM‐identifying individuals —representation is everyone's responsibility	Increased safety and reduced experiences of microaggressions Reduced feelings of the ‘minority tax’ upon SGM individuals Surfacing of issues in real time with quality improvement in experiences of belonging	Distribution and coordination of responsibility across institutional bodies Collectively role modelling a culture of belonging at an institutional level

*Note*: Each intervention suggestion is given alongside the themes with which they most closely align, and associated issues we identified through our research, at the intersection of SGM and URM identities.

### 4.4 Limitations and future directions

Traditionally, URM identity research tends to focus on ethnicity and race.^20^, ^41^, ^49^, ^61^, ^68^, ^70^, ^71^, ^72^, ^73^, ^74^ Our exploration of wider URM identities, and inclusion of participants from across the UK, has allowed us to begin to appreciate and delineate the heterogeneous experiences SGM medical students from a range of backgrounds and contexts hold. Furthermore, all participants felt our analyses spoke to their individual experiences during contemporaneous and retrospective member checking, suggesting that we have encapsulated narratives across heterogeneous identities without being overly reductionist in our approach.

Our work did have some limitations and associated areas for future work. Despite advertisement to all year groups, participants mainly fell within later year categories, perhaps because they felt more settled in how their SGM identities existed within their medical student identity. Additionally, we anticipate that students who felt relatively comfortable in their SGM identities were unavoidably more likely to participate despite our recruitment materials reassuring of anonymity. Taken together, our results may speak to those who are more socially open in their SGM identities. Furthermore, our results underline how cultural, social and political landscapes can influence identity disclosure and role modelling relationships. Only one participant (MS25) was interviewed following legislative changes around the definition of women in the UK, which they mentioned in their narrative. Exploring how our results may be upheld temporally within the shifting political landscape could add greater nuance to our findings. Finally, exploration of how role modelling continues to be important beyond qualification into postgraduate settings is relatively unexplored.^19^ Many participants in our study highlighted that clinical placements were often where role modelling became most evident. Exploring the phenomenon from both ends of the hierarchy by examining clinicians' experiences could provide powerful insights into the driving and hindering forces behind role modelling relationships in the clinical setting.

## 5 CONCLUSION

Role models socialise medical students into the ‘culture and norms’ of medicine^75^ − norms that largely prioritise cisgendered and heterosexual identities. With an intersectional approach, we were able to begin to examine the influence that societal power exerts over personal identities and understand how role modelling may be used to challenge these pervasive norms. Representation is important not only in visible positions of power, but also occurs by giving voice to URM medical students within truly safe and supportive environments. Empowered students mean empowered potential role models themselves, aiding in dismantling the power incongruencies that hinder positive role modelling relationships from both ends of the institution–student balance.

## AUTHOR CONTRIBUTIONS

**Antony P. Zacharias:** Investigation; funding acquisition; writing—original draft; methodology; writing—review and editing; formal analysis; project administration; validation. **Robert Douglas:** Investigation; funding acquisition; writing—original draft; methodology; validation; writing—review and editing; formal analysis. **Debbie Aitken:** Data curation; supervision; investigation; funding acquisition; writing—review and editing; validation; methodology; formal analysis; project administration.

## CONFLICT OF INTEREST STATEMENT

We declare no conflicts of interest for all authors.

## ETHICS STATEMENT

This project was granted ethical approval by the University of Oxford Department of Education Departmental Research Ethics Committee on 28th January 2025 (DREC **–** 1 013 856). The project was also reviewed by the UK Medical Schools Council Education Leads Advisory Group and approved for circulation by education leads in UK Medical Schools.

## Supporting information


**Appendix S1:** Interview schedule used for semi‐structured interviews.

**Appendix S2:** Group experiential themes and associated subthemes, reflecting commonalities and differences in experiences between participants visually.

## Data Availability

The data that support the findings of this study are available on request from the corresponding author. The data are not publicly available due to privacy or ethical restrictions.
