# Supplementary material for: ‘See me for me’: An intersectional approach exploring sexual and gender minority medical students' experiences of role models
Source: Med Educ. 2026 Feb 10;60(9):987–1000. doi: 10.1111/medu.70185 (PMC13441201; doi:10.1111/medu.70185)
Supplement: Supplementary file 1 [file MEDU-60-987-s001.docx]

**Appendix 2** – Group experiential themes and associated subthemes, reflecting commonalities and differences in experiences between participants visually. A tick indicates a strong narrative within the theme; an empty box indicates the theme was not explicitly present.

| **Theme 1: Identity fragmentation, conflict, negotiation and inauthenticity** | MS1 | MS2 | MS3 | MS5 | MS8 | MS9 | MS13 | MS15 | MS25 | MS33 |
| --- | --- | --- | --- | --- | --- | --- | --- | --- | --- | --- |
| A nexus of identities and self-authenticity | ✓ |  | ✓ | ✓ | ✓ | ✓ | ✓ | ✓ | ✓ | ✓ |
| Inauthenticity and internal identity conflict |  |  |  | ✓ | ✓ | ✓ | ✓ |  | ✓ |  |
| Towards an authentic self | ✓ |  |  | ✓ | ✓ |  | ✓ |  | ✓ |  |

| **Theme 2: Negotiating a sense of belonging at the margin** | MS1 | MS2 | MS3 | MS5 | MS8 | MS9 | MS13 | MS15 | MS25 | MS33 |
| --- | --- | --- | --- | --- | --- | --- | --- | --- | --- | --- |
| Compounding experiences of ‘otherness’ | ✓ | ✓ |  | ✓ | ✓ | ✓ | ✓ |  | ✓ |  |
| Shared minority experiences in role modelling relationships | ✓ | ✓ | ✓ | ✓ | ✓ | ✓ | ✓ | ✓ | ✓ | ✓ |
| Defining new margins: communities and belonging | ✓ | ✓ | ✓ | ✓ | ✓ | ✓ | ✓ | ✓ | ✓ | ✓ |

| **Theme 3: Making it ‘their’ problem: carrying the minority burden** | MS1 | MS2 | MS3 | MS5 | MS8 | MS9 | MS13 | MS15 | MS25 | MS33 |
| --- | --- | --- | --- | --- | --- | --- | --- | --- | --- | --- |
| Effort and uncertainty within normative contexts | ✓ | ✓ | ✓ | ✓ | ✓ | ✓ | ✓ | ✓ | ✓ | ✓ |
| Towards alleviating the minority burden |  | ✓ |  |  | ✓ | ✓ | ✓ | ✓ | ✓ | ✓ |
| Visibility and possibility in unburdening identities | ✓ | ✓ | ✓ | ✓ | ✓ | ✓ |  |  | ✓ |  |

| **Theme 4: Identity ownership and the path to self-actualisation** | MS1 | MS2 | MS3 | MS5 | MS8 | MS9 | MS13 | MS15 | MS25 | MS33 |
| --- | --- | --- | --- | --- | --- | --- | --- | --- | --- | --- |
| Normativity and identity control | ✓ | ✓ | ✓ | ✓ | ✓ | ✓ | ✓ | ✓ | ✓ | ✓ |
| Contextual agency in reclaiming identity ownership | ✓ |  | ✓ | ✓ | ✓ | ✓ | ✓ | ✓ | ✓ |  |
| Empowerment, activism and disruptive visibility | ✓ | ✓ | ✓ | ✓ | ✓ | ✓ | ✓ | ✓ | ✓ | ✓ |
