# Supplementary material for: ‘See me for me’: An intersectional approach exploring sexual and gender minority medical students' experiences of role models
Source: Med Educ. 2026 Feb 10;60(9):987–1000. doi: 10.1111/medu.70185 (PMC13441201; doi:10.1111/medu.70185)
Supplement: Supplementary file 2 [file MEDU-60-987-s002.docx]

**Appendix 1** – Interview Schedule

- What does your sexual and/or gender minority identity mean to you?
- How do you feel about the way your SGM is projected to others in medical school currently?
  - Are you happy with this?
  - Do you wish it looked any different? If so, what stops you from projecting it in the way that you want?
- How does the medical school environment influence the way you project your SGM identity?
- How do your other minority identities (as identified in the recruitment survey) play a part in your experiences at medical school?
- Does your SGM identity interact with your other minority identities?
  - Inside medical school
  - Outside of medical school
  - Do they affect your sense of belonging / identity expression / experiences?
- IMPLICIT: Do you have anyone at medical school that makes you feel more comfortable in showing your SGM identity?
  - Do they hold the same minority identities as you?
- What you understand by an SGM ‘role model’ at medical school?
- EXPLICIT: Do you have any role models in relation to your SGM identity at medical school?
  - If only identifies faculty, probe if any other students have been role models for them?
- How / why have you selected these role models?
- Does having the same intersectional identities as your role models matter to you (if applicable)?
- Do you have role models with the same intersectional identity outside of medical school (if applicable)?
- Would having role models with the same intersectional identity in medical school would be helpful for you or not (if applicable)?
- Can you think of any examples or times where you made an impact on an SGM student? Did they hold the same minority identities as you?
- Do you consider yourself a role model for SGM people at medical school?
  - Do they hold the same minority identities as you?

Is there anything I haven’t asked or covered that when you read the title of the project, you wanted to mention?
